# Toxic epidermal necrolysis: a retrospective analysis of 17 cases from Central Tunisia

**DOI:** 10.11604/pamj.2014.19.269.3987

**Published:** 2014-11-11

**Authors:** Chaker Ben Salem, Atef Badreddine, Omar Belajouza, Colandane Belajouza, Najet Ghariani, Hmouda Houssem

**Affiliations:** 1 Department of Clinical Pharmacology, Faculty of Medicine of Sousse, Tunisia; 2 Dermatology Ward, Farhat Hached University Hospital, Sousse, Tunisia; 3 Medical Intensive Care, Medical Intensive Care Unit, Sahloul University Hospital, Sousse, Tunisia

**Keywords:** Toxic epidermal necrolysis, Lyell′s syndrome, drug eruption

## Abstract

Toxic epidermal necrolysis (TEN), also known as Lyell's syndrome, is a rare, life-threatening and wide-spread exfoliative disease of the skin and mucous membrane that is most commonly drug-induced. We retrospectively reviewed the charts of 17 patients who suffered from TEN in Farhat Hached University Hospital, Sousse, Tunisia over a 19-year period from January 1994 to January 2013. Causality for suspected drugs was assessed by means of the Naranjo adverse drug reaction (ADR) probability scale. Antibiotics, mainly ß-lactams, were the most common implicated drugs, followed by nonsteroidal anti-inflammatory drugs, anticonvulsants, and allopurinol. The interval between drug intake and onset of the first symptom ranged between few hours and 19 days with a mean period of 6.11 days. There was extensive skin detachment, averaging 66.17% of total body surface area (range 40-95%). The most major complication was infection, occurring in 9 patients (53%). Seven patients died with a mortality rate of 41%.

## Introduction

Toxic epidermal necrolysis (TEN), also known as Lyell's syndrome, is a rare, life-threatening and wide-spread exfoliative disease of the skin and mucous membrane that is most commonly drug-induced [1]. The reported incidence for TEN varies from 0.4 to 1.9 cases per million inhabitants per year [2, 3]. In the majority of cases, drugs are assumed or identified as the main cause of TEN. The most frequently implicated drugs are antibiotics, anti-convulsants, non-steroidal anti-inflammatory drugs (NSAIDs) and allopurinol. Little work has been carried out in Tunisia on the epidemiology of the most serious skin reaction—TEN and was limited to case reports. Hence, the present study was conducted to present the local epidemiology, clinical features, aetiology and complications of this disorder in a University Hospital in Tunisia during a 19-year-period.

## Methods

We retrospectively reviewed the medical charts of 17 patients who suffered from TEN in Farhat Hached University Hospital, Sousse, Tunisia over a 19-year period from January 1994 to January 2013. Diagnosis of TEN was based on clinical features with an epidermal detachment of more than 30% of TBSA and involvement of one or more mucosal surfaces[4]. In 7 patients, diagnosis was also confirmed histologically revealing wide spread necrotic epidermis involving all layers and dermoepidermal separation on skin biopsy. The causality of TEN with suspected drugs was assessed by means of the Naranjo adverse drug reaction (ADR) probability scale [5]. This scale classifies ADRs in four categories, namely, ‘doubtful’, ‘possible’, ‘probable’ and ‘highly probable’. The SCORTEN index, an independently validated predictor of mortality, was also calculated [6].

## Results

Seventeen cases of TEN were analyzed in this study, 14 females and 3 males. Age ranged between 14 and 78 years. Mean age was 42.9 years. Male to female ratio was 0.21. Prodromal symptoms and signs included macular exanthem (*n* = 11), flu-like illness (n = 5), fever (*n* = 4), pruritus (*n* = 5), vomiting (*n* = 2), sore throat (*n* = 2). Progression of symptoms was variable. Diagnosis of TEN was made within 4.23 days from the first symptom. Cutaneous findings on admission were denudation (*n*= 13), bullae (*n* = 7), mucositis (*n* = 17) and purpura (*n* = 3). Nikolsky sign was positive in 14 patients. There was extensive loss of skin, averaging 66.17% of TBSA range: 40% to 95%). In Fifteen patients, detachment represented 60% or more of TBSA (Table 1). All patients in the acute stages had involvement of either oral (*n* = 16), ocular (*n* = 12) or genital (*n* = 11) mucosa.

**Table 1:** Patients characteristics

Case N°	Sex/age (years)	Cause	Naranjo scale	Onset, days	SCORTEN	Maximum Skin detachment
1	F/55	Streptomycin	10: highly probable	4	3	70
2/**D**	F/19	Allopurinol	6: probable	19	2	60
3/**D**	F/65	Ketoprofen	6: probable	3	3	90
4	F/27	Lincomycin	2: possible	8h	2	65
5	M/34	Ampicillin	6: probable	1	3	60
6/**D**	F/64	Ampicillin, gentamicin, ofloxacin	3: possible	2	3	60
7	M/48	Sulfasalazine	2: possible	12	2	95
8	F/24	Acetylsalicyclic acid	3: possible	1	2	80
9	F/14	Unknown			1	60
10	F/19	Paracetamol	5: probable	10h	1	45
11	M/48	Phenobarbital	4: possible	1	3	40
12/**D**	F/34	Trimethoprim-sulfamethoxazole	4: possible	19	4	80
13/**D**	F/47	Phenobarbital	4: possible	2h	2	70
14	F/60	Trimethoprim-sulfamethoxazole	4: possible	7	2	60
15/**D**	F/71	Allopurinol	4: possible	3	2	60
16/**D**	F/78	Amoxicillin/clavulanate	4: possible	15	3	60
17	F/22	Celecoxib	4: possible	10	2	70

**D**: patient died, h: hour

No cause was ascertained in one patient (N°9). In contrast, 16 TEN cases had received drugs and were suspected to be caused mainly by drugs. Using the Naranjo probability scale for causality assessment, 11 ADRs were identified as possible, 4 as probable, and 1 as highly probable (Table 1). Antibiotics (7/17, 41.2%) were the most common implicated drugs, followed by NSAIDs (3/17, 17.6%), anticonvulsants (2/17, 11.7%), and allopurinol (2/17, 11.7%). Beta-lactam antibiotics (3 cases) were the most frequent causative drugs among antibiotics. Rechallenge with the suspected agent was not performed in the majority of cases for ethical reasons because of the risk of a serious relapse, except in a problematic case of a 55-year-old woman with active pulmonary tuberculosis (TB) who developed TEN in the fourth day of standard oral anti-TB chemotherapy including isoniazid, rifampin, pyrazinamide, and streptomycin (Patient n°1). All anti-TB drugs were stopped, but one week later, hemoptysis recurred with fever and evidence of increased cavitations on chest X-ray. Considering the benefit-risk ratio, a decision was made to give her a rechallenge test for anti-TB drugs. A staged-fashion exposure test to the 4 anti-TB drugs led to the conclusion that streptomycin was the causative agent of TEN. The duration between the drug intake and the onset of the first symptom ranged between few hours and 19 days with a mean time-period of 6.11 days.

Many patients showed organ involvement and other complications. Twelve patients (70.5%) had ocular complications. The initial symptoms included conjunctivitis ranging from mild conjunctival injection to purulent conjunctivitis with subsequent pseudomembrane formation (7/17, 41.1%), dry eye (2/17, 11.7%) and ocular pain (2/17, 11.7%). A superficial keratitis was found in one patient, and palpebral necrosis in two patients. Eleven patients had genital erosive vulvovaginitis and one patient developed erosive balanitis and scrotal skin desquamation.

The most serious complication was infection, observed in 9 patients (53%) and including cutaneous infection, pulmonary infection, septicemia and septic shock. Respiratory disorders were seen in 9 cases. They included dyspnea, pneumonia, and acute respiratory failure. Endotracheal intubation was required for 7 patients. Encephalopathy and gastro-intestinal disorder were also observed. Other complications during admission included brainstem ischaemia and junctional tachychardia. Six cases had renal dysfunction. Hemodialysis was performed in one case. Among the 17 patients of this series, 10 had anemia, 5 had thrombocytopenia and 2 had leucopenia. Rhabdomyolysis was reported in 12 cases. Eight patients developed liver cytolysis.

Treatment was supportive in most cases without corticosteroids. The decision of steroid therapy was based on the dermatologist′s own view rather than on the severity of illness. In our study, the SCORTEN was calculated for all patients (median SCORTEN 2.35, range 2-4). Predicted mortality by SCORTEN was 22% (3.75 deaths), whereas observed mortality which 41% (7 deaths).

In this study, seven patients died with a mortality rate of 41% (Table 2). Of the 12 patients with ocular involvement, 4 developed long-term complications, 4 had photophobia, 1 had trichiasis and cicatricial entropion, 2 had xerophthalmia and 1 case developed corneal ulceration and underwent amnion transplantation. The skin loss of all patients healed spontaneously without the need for skin transplantation. One patient with genital involvement had vulvar synechiae requiring restorative surgery.

**Table 2:** Cause of death in patients with TEN

Patient N°	Cause of death	Associated medical conditions
2	Septic shock, respiratory failure, Acinetobacter pneumonia, acute renal failure and metabolic acidosis	Bilateral nephrolithiasis, adiposis
3	Cardiogenic shock, multiple organ failure, metabolic acidosis	Diabetes mellitus, hypertension, right nephrectomy, chronic renal
6	Acute respiratory failure, acute arterial pulmonary hypertension	Hypertension, coronary heart disease
12	Brainstem stroke	Acalculous cholecystitis
13	Septic shock, nosocomial skin infection	Epilepsy, diabetes mellitus, adiposis
15	Septic shock, respiratory failure, pneumonia	Hypothyroidism, gout
16	Septic shock, respiratory failure, nosocomial pneumonia	Cerebral infarction

## Discussion

TEN is a rare disease with an incidence ranging from 0.4 to 1.9 cases per million inhabitants per year [2, 3]. In our series, there were only 17 cases of TEN over a 19-year period. These cases have been observed in only one university hospital of central Tunisia, therefore this fact underestimates the true incidence of TEN. More than half of the patients presented with one or more prodromal symptoms such as fever, flu-like illness, pruritus, sore throat and vomiting. Clinical features of patients with TEN in our series showed that all patients in the acute stage had involvement of either oral (n = 16), ocular (*n* = 12) or genital (*n* = 11) mucosa as described in the literature [7]. Liver involvement with transient elevation of liver enzymes, and renal involvement with elevation of Blood urea nitrogen and serum creatinine were noted in the present study. Hematological abnormalities are a common finding in our study, but it is not known whether there is direct and specific bone-marrow pathology or if it represents a secondary phenomenon. Increased serum creatine kinase was interestingly the most common observed biochemical abnormality. The exact relationship between TEN and rhabdomyolysis is not completely understood but could be explained by an associated myositis.

TEN is an acute disease that is almost always drug related. Our study showed that the aetiology of TEN for 16 out of 17 patients was apparently drug related, and the commonest drugs triggering TEN were ß-lactam antibiotics. This is in concordance with the results of the study done in West Germany which incriminated antibiotics in 42% of TEN cases [8]. Antibiotics, mainly beta-lactams, were the most common antibiotics causing Stevens–Johnson syndrome-TEN in a study by Wong et al [9]. This is probably due to their more frequent use compared with other antibiotics. Only one patient in our series developed TEN due to streptomycin. Streptomycin-induced TEN is an extremely rare adverse drug reaction. A cause–effect relationship is difficult to establish when multiple drugs, known as TEN inducers, are concomitantly prescribed. However, considering the benefit-risk ratio, the rechallenge test for anti-TB drugs concluded a highly probable Naranjo score for streptomycin. Several Indian studies have shown anticonvulsants as the commonest causative drugs for TEN [10, 11]. In Europe, allopurinol is the most common cause of TEN [12]. The mean reaction time between ingestion of the drug and onset of symptoms for our patients was 6.11 days. The majority of symptoms manifested within the first week of drug intake. According to the EuroSCAR study, an interval of 4 to 28 days is highly suggestive of drug causality in TEN [13].

The average reported mortality rate of TEN is 6.2-36% [8, 14]. In our study, the SCORTEN was inaccurate in predicting mortality and there was a bad correlation between predicted and observed mortality at each score level and overall. Seven patients died with a mortality rate of 41%. They had an average of 68.5% TBSA detached skin. Most of the patients who died had both severe underlying disease and increased mean age (54 vs. 35.1 years for the 10 patients who survived). The SCORTEN index does not take into account chronic illness, and seems to underestimate the risk of death as appeared in our series. The observed mortality rate in our study is higher than in other published series. This might be explained by the fact that most of the TEN patients had extensive skin necrosis and therefore had more complications.

## Conclusion

TEN is an acute disease that is almost always drug related. Antibiotics, mainly ß-lactams followed by NSAIDs, anticonvulsants, and allopurinol were the main offending agents in our study. The observed mortality rate in our study is higher than in other published series. Long-term complications, especially ocular sequelae, were an important cause of TEN associated morbidity.
